# Endoscopic ultrasound-guided mediastinal mass biopsy in an undiagnosed pediatric liver transplant recipient

**DOI:** 10.1055/a-2336-3197

**Published:** 2024-06-25

**Authors:** Giacomo Emanuele Maria Rizzo, Mario Traina, Dario Ligresti, Lucio Carrozza, Gabriele Rancatore, Giusy Ranucci, Ilaria Tarantino

**Affiliations:** 1 18326 Endoscopy Service, Department of Diagnostic and Therapeutic Services, ISMETT, Palermo, Italy; 2 18998 Precision Medicine in Medical, Surgical, and Critical Care, University of Palermo, Palermo, Italy; 3 18326 Pediatric Unit, ISMETT, Palermo, Italy

A 6-year-old girl with biliary atresia underwent a left-sided partial liver transplantation at the age of 1 year, starting immunosuppressive therapy thereafter. She developed portal cavernoma after liver transplantation, which was not treated by shunt meso-rex or interventional radiology. At the age of 5 years, she developed mediastinal lymphadenopathy and pleural effusion due to thoracic duct leak (chylothorax), necessitating bilateral pleurodesis, which resolved the decompensation. After a few months without symptoms, she developed respiratory insufficiency, fever, and cough, requiring oxygen therapy.

Computed tomography showed enlarged mediastinal lymph nodes, multiple abdominal lymph nodes, thickening of pulmonary interlobular septa, ground-glass opacities in the pulmonary parenchyma, bilateral large pleural effusions, and ascites with signs of portal hypertension (esophageal varices, collateral vessels, and splenomegaly) ( Fig. 1 ). Laboratory findings revealed elevated erythrocyte sedimentation rate and other acute phase reactants, anemia, leukopenia, eosinophilia, hypergammaglobulinemia, and impaired delayed hypersensitivity on skin testing.

**Fig. 1:**
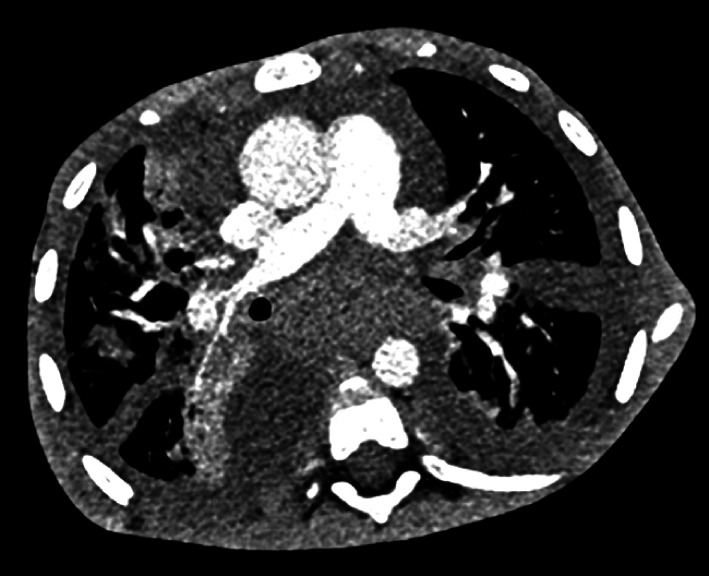
Computed tomography showing mediastinal lymphadenopathy and pulmonary changes in a 6-year-old liver transplant recipient.

A double infection with rhinovirus and pneumococcus involving abdominal and mediastinal lymph nodes was suspected. Considering the negative autoimmune antibody tests, a multidisciplinary board meeting was conducted, in which the decision was made to perform endoscopic ultrasound (EUS) to obtain tissue specimens from the mediastinal masses for histological and microbiological analyses.

Pediatric EUS-guided tissue acquisition (EUS-TA) of mediastinal masses is challenging and difficult. It is rarely performed even in tertiary centers, particularly in patients receiving immunosuppressive therapy and those with a large pleural effusion. Moreover, a standard EUS scope is excessively large for a 6-year-old girl with malnutrition. Therefore, we decided to perform transesophageal EUS with bronchoscope-guided fine-needle biopsy (EUS-B-FNB) 1 ( Fig. 2 , Video 1 ) to prevent injury due to the large EUS scope. EUS-B-FNB showed multiple hyperechoic mediastinal masses and pleural effusion. Therefore, we obtained tissue samples for histological and microbiological examinations using three passages of a 22-G needle. The results revealed infiltration of lymphatic cells without atypical or malignant features in tissues.

**Fig. 2:**
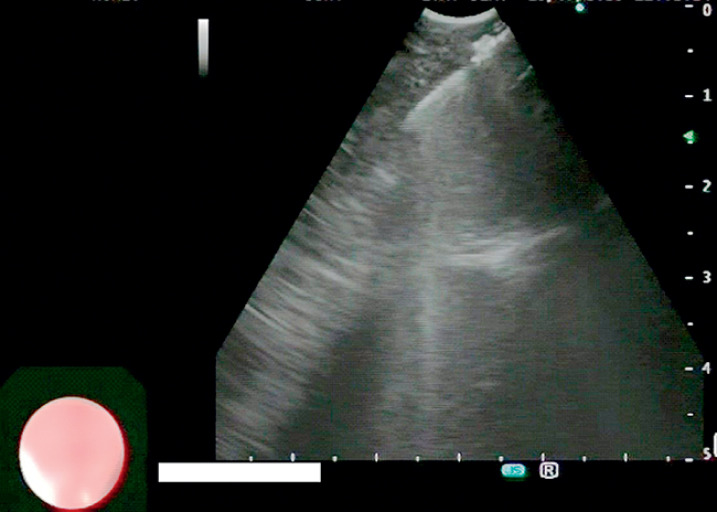
Transesophageal endoscopic ultrasound image with bronchoscope-guided fine-needle biopsy in a 6-year-old liver transplant recipient.

Transesophageal endoscopic ultrasound with bronchoscope-guided fine-needle biopsy of mediastinal lymph nodes in a 6-year-old girl with a history of biliary atresia and liver transplant.Video 1

Extensive diagnostic tests for infection in the blood and tissues were negative. In addition, several empirical antimicrobial treatments were administered without any clinical effects. Further investigations, including bone marrow aspiration and pulmonary scintigraphy, were negative. Due to the worsening clinical condition of the patient and no known infective or hemato-oncologic conditions, high dose steroids were administered (2 mg/kg), resulting in rapid and substantial clinical improvement, resolution of need of oxygen therapy within a few days, and rapid improvement in radiological findings ( Fig. 3 ).

**Fig. 3:**
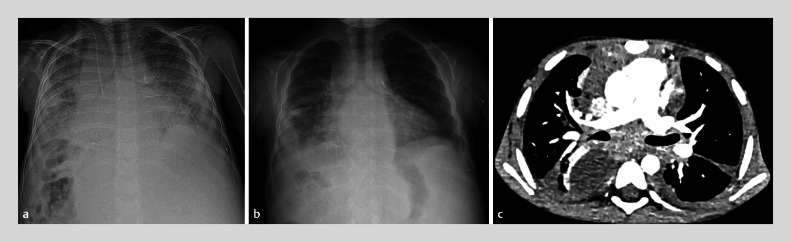
**a, b** Chest radiography before **(a)** and after **(b)** treatment with steroids. **c** Computed tomography of the chest showing improvement after treatment with steroids.

Considering that histological examination of the biopsy specimen from lymph nodes demonstrated typical noncaseating epithelioid cell granulomas in the absence of any evidence of infectious granulomatous conditions or neoplasia, we suspected an autoinflammatory condition related to reticuloendothelial sarcoidosis. The patient was started on immunosuppressive treatment with mycophenolate mofetil and discharged with improved regular pulmonary function. At the 3-month follow-up, she showed improved nutritional status.

This pediatric case demonstrates that EUS-TA is technically feasible and, in some cases, useful for providing fundamental diagnostic information in liver transplant recipients. Importantly, EUS-TA can be extremely useful, as previously mentioned, for the management of life-threatening cases in which malignancies and post-transplant lymphoproliferative disease should be excluded to guide the appropriate treatment.

Endoscopy_UCTN_Code_CCL_1AF_2AC

## Notes

Endoscopy E-Videos https://eref.thieme.de/e-videos
*E-Videos* is an open access online section of the journal *Endoscopy* , reporting on interesting cases and new techniques in gastroenterological endoscopy. All papers include a high-quality video and are published with a Creative Commons CC-BY license. Endoscopy E-Videos qualify for HINARI discounts and waivers and eligibility is automatically checked during the submission process. We grant 100% waivers to articles whose corresponding authors are based in Group A countries and 50% waivers to those who are based in Group B countries as classified by Research4Life (see: https://www.research4life.org/access/eligibility/ ). This section has its own submission website at https://mc.manuscriptcentral.com/e-videos .
